# Associations between noncommunicable disease risk factors, race, education, and health insurance status among women of reproductive age in Brazil — 2011^☆^^☆☆^

**DOI:** 10.1016/j.pmedr.2016.03.015

**Published:** 2016-04-07

**Authors:** Jonetta Johnson Mpofu, Lenildo de Moura, Sherry L. Farr, Deborah Carvalho Malta, Betine Moehlecke Iser, Regina Tomie Ivata Bernal, Cheryl L. Robbins, Felipe Lobelo

**Affiliations:** a Division of Reproductive Health, National Center for Chronic Disease Prevention and Health Promotion, Centers for Disease Control and Prevention, 4770 Buford Hwy, MS-F-74, Atlanta, GA 30341, United States; b U.S. Public Health Service Commissioned Corps, 1101 Wooten Parkway, Rockville, MD 20852, United States; c Pan-Americana Health Organization, Brasilia, Distrito Federal, Brazil; d Ministry of Health of Brazil, Section 2, Lots 05/06, Premium Building, Block F, Tower 1, Brasilia, DF, Brazil; e Hubert Department of Global Health, Rollins School of Public Health, Emory University, 1518 Clifton Road, Mailstop 1518-002-7BB, Atlanta, GA 30322, United States

**Keywords:** Women, Reproductive health, Education, Insurance, Health, Risk factors, Chronic disease

## Abstract

**Background:**

Noncommunicable disease (NCD) risk factors increase the risk of adverse reproductive health outcomes and are becoming increasingly common in Brazil.

**Methods:**

We analyzed VIGITEL 2011 telephone survey data for 13,745 Brazilian women aged 18–44 years in a probabilistic sample from 26 Brazilian state capitals and the Federal District. We examined associations between NCD risk factors (fruit and vegetable intake, leisure time physical activity, alcohol consumption, smoking status, BMI and hypertension status) and race, education, and insurance using chi-square tests and multivariable logistic regression models, estimating the average marginal effects to produce adjusted relative risk ratios (aRRs). Analyses were conducted using SAS 9.3 survey procedures and weighted to reflect population estimates.

**Results:**

Women with less than a college education were more likely to report physical inactivity (adjusted relative risk (aRR) and 95% confidence interval = 1.1 (1.1–1.2)), smoking (aRR = 1.7 (1.3–2.2)), and self-reported diagnoses of hypertension (aRR = 2.0 (1.6–2.5)) compared to women with a college education or greater. Similarly, women without health insurance were more likely to report physical inactivity (aRR = 1.1 (1.1–1.2)), smoking (aRR = 1.4 (1.1–1.8)), and self-reported diagnoses of hypertension aRR = 1.4 (1.1–1.7)) compared to women with health insurance. Less variation was found by race and NCD risk factors.

**Conclusion:**

Targeted public health strategies and policies are needed to increase healthcare access and decrease educational and racial disparities in NCD risk factors among women of reproductive age in Brazil.

## Highlights

• Examine prevalence of NCD risk factors with race, education, and insurance status
• We analyzed data from a probabilistic sample of 13,745 non-pregnant Brazilian women.
• Less educated women reported more physical inactivity, smoking, and hypertension.
• Uninsured women reported more physical inactivity, smoking and hypertension.
• Less variation was found by race and noncommunicable disease risk factors.

## 1 Introduction

Noncommunicable diseases (NCDs), defined as cardiovascular disease, cancer, chronic respiratory diseases, and diabetes, are major causes of morbidity and mortality in low- and middle-income countries (Alwan et al., 2011, Hunter and Reddy, 2013, Roura and Arulkumaran, 2014). In recent reports, the World Health Organization estimates that approximately 42% (16 million) of NCD deaths annually are premature, deaths occurring before age 70. Of the 16 million premature NCD deaths, 82% occur in low- to middle-income countries (World Health Organization, 2013, World Health Organization, 2015). In 2007, approximately 72% of all deaths in Brazil were attributable to NCDs, and racial and ethnic minority groups were disproportionately affected (Schmidt et al., 2011). Rapid increases in urbanization, industrialization, and income over the past several decades in Brazil have contributed to increasing prevalence of NCDs and their risk factors (referred to as NCD risk factors hereafter) (Schmidt et al., 2011).

NCD risk factors include tobacco use, physical inactivity, an unhealthy diet, alcohol abuse, obesity, and hypertension (World Health Organization, 2013). NCD risk factors are shaped by race, economic status, and education, which indirectly shape NCD morbidity and mortality (do Carmo et al., 2005, Linetzky et al., 2013). Additionally, women with NCD risk factors are at higher risk for adverse reproductive health outcomes for themselves and their infants, such as gestational diabetes (Bombard et al., 2012), gestational hypertension (Bombard et al., 2012, Ferrer et al., 2000), pre-eclampsia (Duckitt and Harrington, 2005, Livingston et al., 2003), and increased risk of macrosomia and other negative birth outcomes (Arendas et al., 2008, Begum et al., 2011, Leddy et al., 2008, Roura and Arulkumaran, 2014). Such adverse reproductive health outcomes may also adversely affect offspring later in life (Barker et al., 1989, Bloomfield, 2011, Eriksson, 2005, Roura and Arulkumaran, 2014, Vieau, 2011) and contribute to premature mortality and major cardiovascular events for mothers later in life (Charach et al., 2015, Lee and Tubby, 2015, Lee et al., 2015). The contribution of race and socioeconomic factors such as economic status and education on NCD risk can also influence infant and maternal reproductive health outcomes by patterning access to healthcare and health resources for women of reproductive age (Nagahawatte and Goldenberg, 2008). Little to no research has examined associations between NCD risk factors, race and socioeconomic factors among non-pregnant women of reproductive age in Brazil.

Because of an increasing burden of NCD risk factors in Brazil and their associations with adverse reproductive health outcomes, surveillance is needed to examine characteristics associated with NCD risk factors among non-pregnant women of reproductive age in Brazil (do Carmo et al., 2005). Our objective was to estimate the prevalence of NCD risk factors and their associations with race, education, and health insurance status among non-pregnant women of reproductive age in Brazil.

## 2 Methods

We used 2011 data from Sistema de Vigilância de Fatores de Risco e Proteção para Doenças Crônicas por Inquérito Telefônico (Telephone-based Surveillance of Risk and Protective Factors for Chronic Diseases, or VIGITEL) (Moura et al., 2006). VIGITEL uses probabilistic samples of the adult population (≥ 18 years of age) selected from residential listings of households with telephones in each capital of the 26 Brazilian states and the Federal District. Respondents gave verbal consent at the time of the telephone call. VIGITEL was approved by the National Ethics Committee on Human Research of the Ministry of Health of Brazil (protocol number 355.590/2013). Of 83,401 telephone lines eligible in 2011, approximately 54,000 interviews were performed, for a response rate of 65%. For this analysis, we restricted the sample to women aged 18–44 years who were not currently pregnant (N = 15,301). We excluded 1556 (12.1%) women who responded ‘*don't know*’ or refused to answer questions about racial/ethnic group, education, health insurance status, covariates, or body mass index. Our final study sample consisted of 13,745 women.

The dependent variables, NCD risk factors, were categorized as behavioral or biological. Behavioral NCD risk factors were current smoking (women who answered yes to the question, “Do you smoke?” were considered to be smokers independent of frequency and duration of smoking habit); insufficient leisure time physical activity (< 150 min/week of moderate physical activity or < 75 min/week of vigorous physical activity over the last 3 months); binge drinking (consuming ≥ 4 alcoholic beverages on the same occasion in the past 30 days); and inadequate intake of fruit, legumes, and vegetables (eating < 5 servings/day on ≥ 5 days/week). Biological NCD risk factors were obesity (body mass index [BMI] ≥ 30 kg/m^2^) and self-reported previous medical diagnosis of hypertension. Independent variables were self-reported racial group (white, black, Asian, mixed race, or Native Brazilian), educational level (< college or university education, ≥ college or university education), and health insurance status (uninsured: government free, public national health system only (*Sistema Único de Saúde, SUS*), insured: ≥ 1 private insurance plans). Private insurance plans include private health insurance, prepaid group practice, medical cooperatives and company health plans. Covariates were age in years (18–19, 20–24, 25–29, 30–34, 35–39, 40–44), marital status (unmarried, married), and employment status (unemployed/not worked over past 3 months, employed).

### 2.1 Statistical analysis

Analyses were conducted using SAS 9.3 survey procedures and weighted to reflect population estimates. We calculated weighted prevalence and 95% confidence intervals (CIs) for all demographic characteristics and for behavioral and biological NCD risk factors overall and by racial group and examined associations using chi-squared tests. Additionally, we calculated weighted prevalence and 95% CIs for behavioral and biological NCD risk factors by educational level and health insurance status and examined associations using chi-squared tests. Finally, we examined associations between behavioral and biological NCD risk factors and racial group, education level, and health insurance status in logistic regression models with the average marginal effect statement to produce adjusted relative risk ratios (aRRs) and 95% CIs. We examined associations between racial group, education level and health insurance in a single model to gain clarity on the association of each with NCD risk factors while controlling for the others. All models were adjusted for age, marital status, and employment status. For all analyses, significance was set at P < 0.05. This study was approved by the National Commission for Ethics in Human Research, Brasilia, Federal District, Brazil.

## 3 Results

Of the 13,745 women in the final sample, the majority were unmarried (68.3%), had less than a college or university education (66.2%), and were uninsured (51.4%) (Table 1). About one-third (30.6%) were unemployed or had not worked over the previous 3 months. We found significant differences across racial groups for all demographic characteristics except employment status (Table 1). Mixed-race women had the highest prevalence of less than a college or university education (75.9%; 95% CI 74.0–77.7), while white women had the lowest prevalence (56.6%; 95% CI 54.0–59.1). Black women had the highest prevalence of being uninsured (64.3%; 95% CI 59.6–69.1), while white women had the lowest (41.6%; 95% CI 49.8–53.1).

For the behavioral NCD risk factors, most women had inadequate weekly intake of fruit, legumes, and vegetables (77.3%) and insufficient weekly leisure time physical activity (73.7%). Less than 10% of women were current smokers, and 12% reported binge drinking in the past 30 days (Table 1). Native Brazilian women had the highest prevalence of inadequate intake of fruit, legumes, and vegetables (84.7%; 95% CI 77.5–91.8), while white women had the lowest (75.5%; 95% CI 73.3–77.6). Black women had the highest prevalence of binge drinking (18.1%; 95% CI 13.9–22.3) and Native Brazilian women had the lowest (9.1%; 95% CI 3.3–15.0). For biological NCD risk factors, 11.7% of women were obese, and 11.5% had self-reported diagnosed hypertension. There were no significant differences across racial groups for obesity and self-reported diagnosed hypertension.

There were significant differences among all behavioral and biological NCD risk factors by education level and insurance status, except binge drinking (Table 2). Compared to their counterparts, women with less than a college or university education and uninsured women had higher prevalence estimates of inadequate intake of fruit, legumes, and vegetables, insufficient leisure time physical activity, current smoking, obesity, and self-reported diagnosis of hypertension (Table 2).

In the multivariate logistic regression model, the aRR for binge drinking was 1.6 times higher among black women than white women (Table 3). Women without a college or university education were significantly more likely than women with a college or university education or greater to have inadequate fruit, legume, and vegetable intake (aRR 1.1; 95% CI 1.1–1.1), insufficient leisure time physical activity (aRR 1.1; 95% CI 1.1–1.2), current smoking (aRR 1.7; 95% CI 1.3–2.2), obesity (aRR 1.4; 95% CI 1.2–1.8), and self-reported diagnoses of hypertension (aRR 2.0; 95% CI 1.6–2.5). Uninsured women were more likely to report insufficient leisure time physical activity (aRR 1.1; 95% CI 1.1–1.2), current smoking (aRR 1.4; 95% CI 1.1–1.8), and self-reported diagnosis of hypertension (aRR 1.4; 95% CI 1.1–1.7) compared to women with health insurance (Table 3).

## 4 Discussion

This analysis highlights differences in behavioral and biological NCD risk factors by racial group, education level, and health insurance status among non-pregnant women in Brazil, a country with increasing prevalence of NCD risk factors (Schmidt et al., 2011). With the exception of binge drinking, we found greater variation in risk of NCD risk factors by education level and health insurance status than by racial group. Women with less than a college or university education (compared to college or university-educated) and uninsured women (compared to insured) were more likely to have insufficient leisure time physical activity, be current smokers, and have a self-reported diagnosis of hypertension. Women with less education also were more likely to report obesity and inadequate consumption of fruit, legumes, and vegetables.

Our findings on education and health insurance are similar to those from previous VIGITEL surveys (Iser et al., 2011, Malta and Bernal, 2014). Results from VIGITEL 2009 show that women of all ages with a college or university education had lower prevalence of NCD risk factors than those with less education (Iser et al., 2011). Likewise, an analysis of VIGITEL 2012 showed that adults of all ages with health insurance had a higher prevalence of protective factors, a lower prevalence of NCD risk factors, and better access to preventive health services than those without health insurance (Malta and Bernal, 2014).

While previously published studies using VIGITEL data have examined differences in NCD risk factors by education level and health insurance status (Iser et al., 2011, Malta and Bernal, 2014), only two have examined associations between NCD risk factors and racial group among women ages 18 and older in Brazil (Chor et al., 2004, Malta et al., 2014, Moura and Malta, 2011). Similar to the current analysis, using 2006 VIGITEL data, Moura and colleagues (2011) found that binge drinking was lower among white compared to non-white women (Moura and Malta, 2011). Using 2012 VIGITEL data, unavailable at the initiation of our analysis, adjusted for age and education only, Malta et al. (2014) found that black women aged ≥ 18 years had a lower prevalence of leisure time physical inactivity and higher prevalence of alcohol abuse and hypertension compared to white women, and mixed-race women were less likely to be current smokers compared to white women (Malta et al., 2014). Similar to this analysis but using a different data source, Chor et al. (2004) found that, at study entry, black and mixed-race women aged 30 to 70 years from Rio de Janeiro, Brazil, had higher age-adjusted prevalence of obesity compared to white women (Chor et al., 2004). In our analysis among women of reproductive age, no NCD risk factors other than binge drinking and current smoking varied by racial group in the multivariate logistic regression model. While our associations for physical inactivity, obesity, and hypertension were in similar directions, they did not reach significance. The decreased variation in NCD risk factors by racial group in our study may be due to several factors. Our study focuses on women of reproductive age (18–44 years) unlike other analysis that include women past reproductive age (Chor et al., 2004, Malta et al., 2014) and therefore yields results applicable to women in their childbearing years. Additionally, regression models in the current analysis are adjusted for more factors than Malta et al. (2014), including age, marital status, and employment status, and account for the joint effects of race, education, and health insurance on NCD risk factors by combining them in the same model (Malta et al., 2014). Analysis by Malta and colleagues are only adjusted for age and education (Malta et al., 2014). Fully adjusted regression models in the current analysis may yield more accurate estimates of associations between NCD risk factors, race, education, and health insurance status among women of reproductive age in Brazil by accounting for more variation in variables other analyses leave out. Finally, the current study examines associations between NCD risk factors and black, white, mixed-race, Asian, and Native Brazilian women by modeling race as a nominal variable, whereas analysis by Malta et al. (2014) used a dichotomous race variable (white vs. black or white vs. mixed-race persons) in separate models (Malta et al., 2014). Despite lower significance in associations between racial group and NCD risk factors, it is important to acknowledge race as a variable that may influence education and health insurance. Such unmeasured effects of race may pattern and structure who has access to education and health insurance.

In general, our findings highlight the important roles of education and health insurance in reducing NCD risk factors and resulting NCD outcomes for reproductive-aged women in Brazil. Several factors may underlie the significant role of education and health insurance status in our results. Economic and social development in low- and middle-income countries like Brazil has resulted in increased urbanization, globalization, greater variation in individual socioeconomic status, and increased prevalence of NCD risk factors and resulting NCD outcomes. A policy-centered approach that uses legislature to enforce global governmental agreed upon goals to reduce NCD outcomes and manage NCD risk factors is especially important for women of reproductive age because of the negative consequences of NCD risk factors and NCDs on pregnancy and pregnancy outcomes (Bonita and Beaglehole, 2014, do Carmo et al., 2006, Maina, 2011, The NCD Alliance, 2011). Due to the increased burden of NCD risk factors and NCD outcomes on women in Brazil (do Carmo et al., 2005, do Carmo et al., 2006, Schmidt et al., 2011), it is important to prevent NCD risk factors before a pregnancy (Roura and Arulkumaran, 2014). There has been increased attention focused on integrating NCD screening and counseling on NCD risk factor risk reduction into maternal and child health programs (Svitone et al., 2000, Victoria et al., 2011). Such integrated efforts may prove effective in decreasing NCD risk factors among women of reproductive age in Brazil (Gomes et al., 2014, Maina, 2011, Malta and da silva, 2012, Ramos et al., 2014). Policy makers and public health practitioners may want to consider ways to target uninsured and less educated women in these efforts.

One such policy approach that targets uninsured and less educated women is the government free public national health system, SUS. While results from our cross-sectional study using VIGITEL 2011 data show that women without private health insurance had a higher prevalence of certain NCD risk factors, a recent study by Malta et al. (2015) show a decrease in the prevalence of several NCD risk factors among men and women sampled in VIGITEL from 2008–2013 (Malta et al., 2015). In that study, Malta and colleagues also found an increase in the prevalence of uninsured women (without private health insurance) with access to mammogram screening (Malta et al., 2015). Such results are evidence that while women without private health insurance historically have less access to preventive health services, implementation of SUS and other services has been successful at attenuating economic and social inequalities in access to preventive health services and increasing the prevalence of protective NCD behaviors.

### 4.1 Limitations

This analysis has several limitations. First, the study was limited to women in Brazil living in each capital of the 26 Brazilian states and the Federal District with access to a residentially listed landline telephone. Limiting the sample to homes in capital cities with landline telephones may exclude women of lower socioeconomic status who cannot afford a landline telephone, those who live in more rural areas, or younger women who may be of higher socioeconomic status but less frequently have a landline. VIGITEL weighting factors were used to adjust telephone survey estimates to correct for differences in the Brazilians who do and do not own a landline telephone. All data are self-reported, and women tend to underreport and underestimate their weight, which may lead to underestimation of BMI (Abbot et al., 2008, Tsai et al., 2015). Past research in Brazil showed a tendency for Brazilians of African descent to self-report a race other than African descent (Chor et al., 2004, Skidmore, 1995). However, this practice is less frequent in recent literature and less common in more developed areas (Chor et al., 2004). Misclassification of race may have led to underestimating its association with NCD risk factors. Differences in health insurance status may have also resulted in differential misclassification of self-reported hypertension and led to underestimation of hypertension among uninsured women. Additionally, the use of leisure time physical activity does not capture occupational, household or transportation related activity. The use of leisure time physical activity, however, has been documented as a more reliable indicator of physical activity in Brazil where occupational and household physical activity tend to be over reported (Hallal et al., 2010). In addition, we excluded 12.1% of women who responded ‘don't know’ or refused to answer questions about racial/ethnic group, education, health insurance status, covariates or BMI. Is it unknown if these women were more likely to be non-white or have NCD risk factors examined in this study. Finally, not all women of reproductive age can become pregnant. While efforts to reduce NCD risk factors in non-fecund women will not improve pregnancy outcomes, they can improve women's health.

### 4.2 Conclusion

Focusing public health efforts on reducing the prevalence of NCDs and their risk factors for women of reproductive age before they become pregnant may yield important health outcomes for women and their infants. Results from this analysis support the need for more targeted public health strategies and policies to decrease disparities in NCD risk factors among less educated and uninsured women. Such efforts may ultimately yield long-term improvements in NCD risk factors and related outcomes for women of reproductive age in Brazil.

## Disclosure

The authors report no conflict of interest.

## Acknowledgment of funding

U.S. Centers for Disease Control and Prevention.

## Figures and Tables

**Table 1:** Demographic characteristics and NCD risk factors by race among 13,745 women of reproductive age in Brazil, VIGITEL 2011.

Variables	Total	White	Black	Asian	Mixed race	Native Brazilian	
N = 13.745	N = 5460	N = 1313	N = 512	N = 6269	N = 191	Chi square P-value
Weighted % (95% CI)	Weighted % (95% CI)	Weighted % (95% CI)	Weighted % (95% CI)	Weighted % (95% CI)	Weighted % (95% CI)
*Demographic characteristics*							
Age (years)							**0.03**
18–19	7.3 (6.5–8.1)	7.0 (5.6–8.4)	6.7 (4.5–9.0)	11.7 (7.0–16.4)	7.5 (6.3–8.7)	5.2 (1.2–9.3)
20–24	17.5 (16.2–18.8)	17.9 (15.8–19.9)	16.0 (12.9–19.2)	23.4 (17.1–29.8)	16.8 (14.8–18.8)	21.0 (8.9–33.2)
25–29	20.4 (19.0–21.7)	20.3 (18.0–22.6)	21.3 (17.0–25.5)	18.8 (12.9–24.8)	20.4 (18.5–22.3)	20.4 (9.0–31.7)
30–34	20.7 (19.5–22.0)	20.1 (18.0–22.1)	22.1 (18.1–26.1)	19.7 (14.0–25.5)	20.9 (19.0–22.7)	29.9 (17.7–42.1)
35–39	16.1 (15.0–17.3)	16.8 (14.9–18.7)	16.6 (13.3–20.0)	12.1 (6.4–17.7)	15.7 (14.0–17.3)	13.7 (5.0–22.4)
40–44	18.0 (16.8–19.2)	18.1 (16.2–19.9)	17.2 (13.0–21.3)	14.2 (9.3–19.1)	18.7 (17.0–20.5)	9.7 (4.9–14.5)
Unmarried	68.3 (66.9–69.8)	65.2 (62.7–67.6)	74.4 (70.5–78.4)	72.8 (66.6–79.1)	69.8 (67.8–71.9)	70.8 (58.5–83.1)	**< 0.01**
Unemployed/not worked over past 3 months	30.6 (29.1–32.0)	28.7 (26.5–30.9)	29.1 (24.6–33.6)	31.1 (24.7–37.5)	33.0 (30.8–35.2)	26.3 (16.3–36.3)	0.07
< College or university education	66.2 (64.7–67.8)	56.6 (54.0–59.1)	71.1 (66.6–75.6)	62.4 (54.8–70.0)	75.9 (74.0–77.7)	67.9 (55.4–80.4)	**< 0.01**
Uninsured	51.4 (49.8–53.1)	41.6 (38.9–44.2)	64.3 (59.6–69.1)	50.5 (42.9–58.0)	58.8 (56.5–61.1)	59.5 (46.5–72.5)	**< 0.01**

*Behavioral NCD risk factors*							
Inadequate intake of fruit, legumes & vegetables (< 5 servings/day on ≥ 5 days/week)	77.3 (76.0–78.6)	75.5 (73.3–77.6)	80.4 (76.7–84.0)	80.5 (75.0–86.1)	78.0 (76.1–80.0)	84.7 (77.5–91.8)	**0.04**
Insufficient leisure time physical activity	73.7 (72.4–75.1)	71.8 (69.5–74.0)	73.5 (69.2–77.8)	74.7 (68.8–80.5)	75.6 (73.7–77.5)	80.1 (72.0–88.3)	0.08
Binge drinking (≥ 4 servings/sitting) in past 30 days	12.0 (11.0–12.9)	11.5 (10.0–13.0)	18.1 (13.9–22.3)	9.2 (5.9–12.4)	11.2 (9.8–12.6)	9.1 (3.3–15.0)	**< 0.01**
Current smoker	9.1 (8.0–10.2)	9.6 (7.9–11.4)	10.9 (6.7–15.1)	9.3 (4.6–14.0)	8.1 (6.6–9.6)	9.3 (2.4–16.2)	0.58

*Biological NCD risk factors*							
Obese body mass index (BMI) (kg/m^2^), ≥ 30	11.7 (10.7–12.8)	11.4 (9.8–13.1)	13.7 (10.6–16.7)	9.4 (5.4–13.4)	11.8 (10.3–13.3)	10.6 (4.0–17.3)	0.55
Self-reported diagnosed hypertension	11.5 (10.4–12.5)	10.7 (9.0–12.3)	15.5 (12.0–19.1)	12.1 (6.0–18.3)	11.2 (9.8–12.7)	11.7 (3.3–20.1)	0.11

The bold numbers represent significance at P < 0.05.

**Table 2:** NCD risk factors by education and health insurance status among women of reproductive age in Brazil, VIGITEL 2011.

NCD risk factors	Education level		Health insurance	
< College/university education	≥ College/university education	No	Yes
	Weighted % (95% CI)	Weighted % (95% CI)	Weighted % (95% CI)	Weighted % (95% CI)
*Behavioral*				
Inadequate intake of fruit, legumes & vegetables (< 5 servings/day on ≥ 5 days/week)^a^, ^b^	79.9 (78.3–81.5)	72.2 (69.8–74.6)	80.1 (78.3–81.9)	74.3 (72.4–76.3)
Insufficient leisure time physical activity^a^, ^b^	77.4 (75.8–79.0)	66.5 (64.0–68.9)	79.2 (77.5–81.0)	67.9 (65.8–69.9)
Binge drinking (≥ 4 servings/sitting) in past 30 days	11.2 (10.1–12.4)	13.3 (11.6–15.1)	11.1 (9.8–12.4)	12.9 (11.4–14.3)
Current smoker^a^, ^b^	10.7 (9.2–12.1)	6.1 (4.7–7.5)	11.2 (9.5–13.0)	6.9 (5.6–8.2)

*Biological*				
Obese body mass index (BMI) (kg/m^2^), ≥ 30^a^, ^b^	13.5 (12.1–14.8)	8.3 (6.8–9.7)	13.6 (12.0–15.1)	9.8 (8.5–11.1)
Self-reported diagnosed hypertension^a^, ^b^	14.1 (12.7–15.6)	6.2 (5.0–7.4)	14.2 (12.6–15.8)	8.5 (7.3–9.8)

a Chi square P-value < 0.05 for difference in NCD risk factors or outcome by education level.

b Chi square P-value < 0.05 for difference in NCD risk factors or outcome by health insurance status.

**Table 3:** Behavioral and biological NCD risk factors by race, educational level, and health insurance status among women of reproductive age in Brazil, VIGITEL 2011.

	Inadequate fruit, legume & vegetable intake	Insufficient leisure time physical activity	Binge drinking	Current smoker	Obese body mass index	Self-reported diagnosed hypertension
aRR^a^ (95% CI)	aRR^a^ (95% CI)	aRR^a^ (95% CI)	aRR^a^ (95% CI)	aRR^a^ (95% CI)	aRR^a^ (95% CI)
*Self-reported race*						
White	Ref	Ref	Ref	Ref	Ref	Ref
Black	1.0 (1.0–1.1)	1.0 (0.9–1.1)	**1.6 (1.2–2.1)**	0.9 (0.6–1.4)	1.1 (0.9–1.5)	1.3 (1.0–1.7)
Asian	1.1 (1.0–1.1)	1.0 (0.9–1.1)	0.8 (0.5–1.1)	0.9 (0.5–1.5)	0.8 (0.5–1.2)	1.1 (0.7–1.9)
Mixed race	1.0 (1.0–1.1)	1.0 (1.0–1.1)	1.0 (0.8–1.2)	**0.7 (0.6–0.9)**	0.9 (0.8–1.2)	0.9 (0.8–1.1)
Native Brazilian	1.1 (1.0–1.2)	1.1 (1.0–1.2)	0.8 (0.4–1.6)	0.8 (0.4–1.8)	0.9 (0.5–1.7)	1.0 (0.5–2.1)

*Education level*						
≥ College or university education	Ref	Ref	Ref	Ref	Ref	Ref
< College or university education	**1.1 (1.1–1.1)**	**1.1 (1.1–1.2)**	0.9 (0.8–1.1)	**1.7 (1.3–2.2)**	**1.4 (1.2–1.8)**	**2.0 (1.6–2.5)**

*Health insurance*						
Yes	Ref	Ref	Ref	Ref	Ref	Ref
No	1.0 (1.0–1.1)	**1.1 (1.1–1.2)**	0.8 (0.7–1.0)	**1.4 (1.1–1.8)**	1.2 (1.0–1.5)	**1.4 (1.1–1.7)**

aRR: adjusted relative risk ratio; CI: confidence interval.

a Model includes self-reported race, education level, health insurance, age, marital status and employment status.
